# Simultaneous EEG-fMRI brain signatures of auditory cue utilization

**DOI:** 10.3389/fnins.2014.00137

**Published:** 2014-06-04

**Authors:** Mathias Scharinger, Björn Herrmann, Till Nierhaus, Jonas Obleser

**Affiliations:** ^1^Max Planck Research Group “Auditory Cognition,” Max Planck Institute for Human Cognitive and Brain SciencesLeipzig, Germany; ^2^Department of Neurology, Max Planck Institute for Human Cognitive and Brain SciencesLeipzig, Germany

**Keywords:** audition, categorization, cue weighting, spectro-temporal information, alpha suppression, attention

## Abstract

Optimal utilization of acoustic cues during auditory categorization is a vital skill, particularly when informative cues become occluded or degraded. Consequently, the acoustic environment requires flexible choosing and switching amongst available cues. The present study targets the brain functions underlying such changes in cue utilization. Participants performed a categorization task with immediate feedback on acoustic stimuli from two categories that varied in duration and spectral properties, while we simultaneously recorded Blood Oxygenation Level Dependent (BOLD) responses in fMRI and electroencephalograms (EEGs). In the first half of the experiment, categories could be best discriminated by spectral properties. Halfway through the experiment, spectral degradation rendered the stimulus duration the more informative cue. Behaviorally, degradation decreased the likelihood of utilizing spectral cues. Spectrally degrading the acoustic signal led to increased alpha power compared to nondegraded stimuli. The EEG-informed fMRI analyses revealed that alpha power correlated with BOLD changes in inferior parietal cortex and right posterior superior temporal gyrus (including planum temporale). In both areas, spectral degradation led to a weaker coupling of BOLD response to behavioral utilization of the spectral cue. These data provide converging evidence from behavioral modeling, electrophysiology, and hemodynamics that (a) increased alpha power mediates the inhibition of uninformative (here spectral) stimulus features, and that (b) the parietal attention network supports optimal cue utilization in auditory categorization. The results highlight the complex cortical processing of auditory categorization under realistic listening challenges.

## Introduction

The interpretation of acoustic signals is an essential human skill for goal-directed behavior and vocal communication. The core process underlying this skill—auditory categorization—has been shown to be highly flexible and adaptive, and allows, for instance, speaker recognition in a cocktail party situation (Zion Golumbic et al., 2013), or speech comprehension in noise (Nahum et al., 2008). In both cases, attention has to be directed to the most informative aspect of the acoustic signal (Hill and Miller, 2010).

Neurophysiological studies have suggested that the relative weighting of information during categorization (*information gain* or *cue weighting*, cf. Holt and Lotto, 2006) may be subserved by the interplay between excitatory and inhibitory mechanisms (Thut et al., 2006; Rihs et al., 2007; Weissman et al., 2009). One promising neurophysiological marker of functional inhibition processes are brain oscillations recorded using electroencephalography (EEG), predominantly in the alpha frequency range (8–13 Hz, Foxe et al., 1998; Foxe and Snyder, 2011; Weisz et al., 2011, 2013; Klimesch, 2012). Initially, alpha power had been interpreted as reflecting the degree to which primary cortical areas are in an “idling” mode (Adrian and Matthews, 1934; Niedermeyer and Silva, 2005). More recent studies on auditory comprehension, on the other hand, have shown that the processing of degraded speech stimuli is accompanied by relative decreases in alpha power suppression, i.e., relative increases in alpha power (Obleser and Weisz, 2012; Becker et al., 2013). One interpretation of this finding is that relative increases in alpha power index greater attention and working memory demands under degradation (Ronnberg et al., 2008; Wild et al., 2012). It has been further proposed that brain regions showing high alpha power undergo inhibition, which in turn allows enhanced processing of task-relevant information (Klimesch et al., 2007).

Brain areas underlying the processing and categorization of acoustic information have been identified by means of functional magnetic resonance imaging (fMRI). Previous studies have shown that the posterior part of the superior temporal gyrus (pSTG) is crucially involved in auditory categorization and discrimination (Hall et al., 2002; Guenther et al., 2004; Husain et al., 2006; Desai et al., 2008; Bermudez et al., 2009; Sharda and Singh, 2012). Importantly, in most of these studies, auditory categorization was also subserved by the planum temporale (PT) in the pSTG. The PT has recently received particular attention, because it does not only play a general role in auditory categorization (Griffiths and Warren, 2002; Husain et al., 2006; Obleser and Eisner, 2009) but also a more specific one with regard to the processing of spectral information and pitch (Hall and Plack, 2009; Alho et al., 2014).

Furthermore, feature-selective attentional processes play a crucial role in categorization. Studies concerned with aspects of selective attention during categorization have mainly focused on the visual system (Yantis, 1993; Posner and Dehaene, 1994; Corbetta et al., 2000; Yantis, 2008). These studies identified the inferior parietal lobule (IPL) as an important, hub-like structure, being involved when participants focus attention on informative stimulus features (Shaywitz et al., 2001; Behrmann et al., 2004; Geng and Mangun, 2009; Salmi et al., 2009; Schultz and Lennert, 2009; Gillebert et al., 2012). Existing research on attention in audition has further provided evidence for the involvement of the parietal network (Rinne et al., 2007; Salmi et al., 2009; Hill and Miller, 2010; Henry et al., 2013). In addition, a recent structural imaging (voxel-based morphometry) study also highlighted the role of the IPL in categorization processes (Scharinger et al., 2014).

More recently, the possibility to combine recordings of EEG oscillatory activity and fMRI Blood Oxygenation Level Dependent (BOLD) activity has been explored in several imaging studies. Simultaneous EEG–fMRI recordings (Ritter and Villringer, 2006; Sadaghiani et al., 2010, 2012) suggest that alpha power can be negatively (Goldman et al., 2002; Laufs et al., 2003; Ritter and Villringer, 2006) or positively (Moosmann et al., 2003; Liu et al., 2012) correlated with brain metabolism, depending on the brain regions these correlations are observed in. However, multi-modal neuroimaging evidence on auditory cue weighting during categorization has been essentially absent. Most studies concerned with a functional coupling of alpha power and BOLD signal in selective attention tasks compared the processing of task-relevant information with the processing of task-irrelevant distractor information (e.g., Scheeringa et al., 2012).

It is thus less clear how multiple, potentially competing cues provided by the same acoustic stimulus, will be reflected in alpha-tuned functional processes and concomitant BOLD change. To this end, we designed two stimulus sets for auditory categorization. In the first stimulus set, categorization could be based on spectral properties or physical duration, with spectral properties being more informative. In the second stimulus set, sound duration became the more informative cue, while spectral properties could still be used for categorization. Using combined EEG/fMRI, we asked (a) whether auditory categorization yields a behavioral preference for the most informative stimulus cue in each condition; (b) which brain areas support change in cue utilization, (c) whether alpha power shows relative increases under degradation and (d) whether alpha power correlates with BOLD in brain areas dedicated to the processing of acoustic cues.

## Materials and methods

### Participants

Sixteen healthy volunteers were recruited from the participant database of the Max Planck Institute for Human Cognitive and Brain Sciences (7 females, age range 20–29 years, age 25 ± 2.7 years mean ± standard deviation). They were all right-handed, native speakers of German with no self-reported hearing impairments or neurological disorders. Due to technical problems with EEG acquisition in the magnetic resonance (MR) scanner, we had to exclude one participant from further analyses. Participants gave written informed consent and received financial compensation for their participation. All procedures followed the guidelines of the local ethics committee (University of Leipzig) and were in accordance with the Declaration of Helsinki.

### Stimuli

Stimuli were based on spectral and durational modifications of an inharmonic base signal. This base signal was constructed by adding 16 exponentially spaced sinusoids (ratio between successive components: 1.15) to the lowest sinusoid component frequency of 500 Hz (Goudbeek et al., 2009; Scharinger et al., 2014). We modified the spectral properties of individual sounds by applying a band-pass filter with a single frequency peak, using a second order infinite impulse response (IIR) filter with a bandwidth corresponding to a fifth of its frequency peak. The term “spectral peak” is henceforth used to refer to the filters' center frequency, which also describes the resulting spectral properties. Duration modifications were based on differences in the length of the sounds.

Individual members of category distributions, arbitrarily labeled “A” and “B,” varied on the basis of spectral peak and duration: For individual sounds of each category, spectral filter frequencies and durations were randomly drawn from bivariate normal distributions. These distributions, with equal standard deviations, σ, differed in their means, μ, between the two categories, A and B (Table 1). Thus, each individual sound was characterized by the two dimensions, duration and spectral peak, with means of duration and spectral peak differing between the two category distributions. Each category distribution consisted of 1000 sound exemplars from which a random sample was drawn for each participant in the experiment. Following Smits et al. (2006), we converted spectral peak frequency and duration to scales that allowed for psychoacoustic comparability. Consequently, frequencies were converted to the equivalent rectangular bandwidth (ERB) scale that approximates the bandwidths of the auditory filters in human hearing (Glasberg and Moore, 1990), and durations were converted to a logarithmic scale (DUR; cf. Smits et al., 2006). Table 1 illustrates the means (spectral peak and durations) of the category distributions in psychophysical and physical units.

**Table 1:** **Means and standard deviations (in parentheses) of spectral peak and duration distributions for stimulus categories A and B in the nondegraded and degraded conditions (psychophysical and physical units)**.

**Stimulus category**	**Nondegraded**		**Degraded**	
	**A**	**B**	**A**	**B**
Spectral peak (ERB)	20.00 (0.31)	17.00 (0.31)	16.80 (0.31)	15.50 (0.31)
Spectral peak (Hz)	1739 (8)	1196 (8)	1166 (8)	984 (8)
Duration (DUR)	47.70 (1.31)	52.53 (1.31)	47.70 (1.31)	52.53 (1.31)
Duration (ms)	118 (1.14)	191 (1.14)	118 (1.14)	191 (1.14)

**Figure 1:**
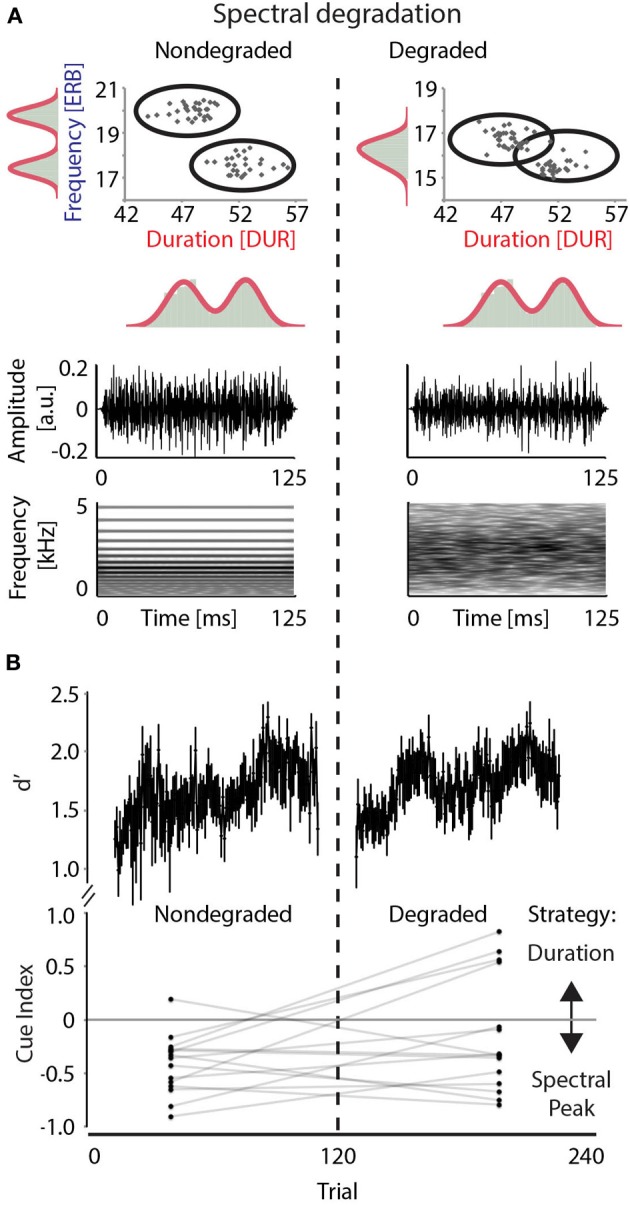
**Stimulus characteristics and behavioral results. (A)** *Top*: Complex sounds differing in spectral peak (expressed in ERB; y-axis) and duration (expressed in DUR; x-axis). Distributions are indicated by ellipses, with black dots illustrating distributions for a representative participant. *Bottom*: Stimulus wave form and spectrogram illustrate the complex structure of sounds in the nondegraded condition (left) and the spectral smearing as a result of vocoding in the degraded condition (right). Duration and amplitude envelope were unaffected by degradation. **(B)** Results of behavioral discrimination. *Top*: Perceptual sensitivity (d′) over time, obtained from sliding windows over nondegraded and degraded trials per participant (window size = 20 trials, step size = 1 trial). *Bottom*: Comparison between by-subject cue indices in the nondegraded and degraded conditions. Mean cue index values for the nondegraded and the degraded conditions are connected for each participant.

In the first half of the experiment (*nondegraded condition*), the two stimulus distributions did not overlap in their spectral peak, but $\frac{1}{3}$ of the sounds in category A and B overlapped in duration (Figure 1A top). This set-up aimed at biasing participants to focus on spectral cues while sound duration may serve as secondary cue. In the second half of the experiment (*degraded condition*), spectral cues were modified by applying four-band noise vocoding to the original stimulus distributions (Drullman et al., 1994; Shannon et al., 1995). Noise vocoding was done by dividing the original signal into four frequency bands, extracting the amplitude envelope from each band and reapplying it to bandpass-filtered noise carriers with matched cut-off frequencies. Envelopes were extracted using a zero-phase, 4th-order Butterworth low-pass filter; the low-pass filter cutoff was set at 256 Hz. Scaling for equal root mean square (RMS) energy was performed channel-wise for each channel envelope (Rosen et al., 1999; Erb et al., 2012). We chose four-band noise vocoding because it offers a well-established reduction of spectrally-based intelligibility (cf. Scott et al., 2006; Obleser and Kotz, 2010; Obleser et al., 2012), thereby ensuring comparability to studies on alpha power suppression in speech, while simultaneously being an ecologically valid modification by simulating effects of cochlear implants (Poissant et al., 2006).

Noise vocoding led to a smearing of spectral detail, while amplitude envelope features and original stimulus duration remained unaffected (Figure 1A, bottom). Thus, as demonstrated before (Scharinger et al., 2014), we aimed at inducing a change in acoustic cue utilization, from spectral peak in the first (nondegraded) condition, to stimulus duration in the second (degraded) condition of the experiment. The stimulus degradation in the second half of the experiment therefore targeted the spectral properties (i.e., spectral peak, but also affected other spectral features such as harmonicity). Thus, degradation of the initially informative spectral cue ought to decrease participants' reliance on that cue and prompt a relatively increased reliance on the duration cue.

All stimuli were normalized for equal root-mean-square intensity and presented at ~60 dB SPL. Onset and offset ramps (5 ms) ensured that acoustic artifacts were minimized.

### Experimental procedure

Participants were first familiarized with the categorization task in the scanner and had to complete a short practice run consisting of 20 sounds (10 from category A and 10 from category B) that did not occur in the main experiment. The subsequent main experiment was arranged in four runs: Two initial runs with nondegraded sounds, and two subsequent runs with spectrally degraded sounds (Figure 1A, top). In each run, 60 sound exemplars, randomly drawn from categories A and B with equal probability, were presented in a sparse imaging design in the MR scanner (Hall et al., 1999). The sparse design was chosen in order to guarantee that stimuli could be presented during silent periods in-between the acquisition of echo-planar images (EPI). At the same time, this design reduced contamination of the EEG signal by gradient switches during volume acquisition.

On each trial, one acoustic stimulus was presented on average 2 s after the offset of a preceding EPI sequence (±500 ms). Subsequently, a visual response prompt (green traffic light) was presented on a screen which participants viewed through a mirror 3 s after stimulus onset. Participants were then required to indicate whether the presented sound belonged to category A or category B by pressing one of two keys on a button box. Button assignment was counterbalanced across participants. Following the response, participants received corrective feedback (*Correct/Incorrect*), which was displayed for 1 s in the middle of the screen. Five seconds after the onset of an acoustic stimulus, a subsequent EPI volume (acquisition time TA = 2 s) was acquired, such that the BOLD peak would best capture stimulus processing. At random positions within each run, 15 silent trials (=20% of all trials) without required responses served as baseline. The duration of the entire experiment with short breaks between runs was 50 min.

### Acquisition and pre-processing of EEG data

The continuous EEG was recorded inside the MR-scanner from 31 Ag–AgCl electrodes mounted on an elastic cap according to the 10–20 standard system (EasyCap-MR, Brain Products, Munich, Germany). The electrocardiogram (ECG) was registered with an additional electrode on the sternum. EEG signals were amplified with an MR-conform 32-channel amplifier (BrainAmp MR; Brain Products, Munich, Germany) that did not get saturated by MR activity. Signals were recorded at a sampling frequency of 5000 Hz and a resolution of 16 bits, referenced against FCz, using the BrainVision Recorder Software (Brain Products, Munich, Germany). The ground electrode was positioned between Fz and FPz. All impedances were kept below 5 kΩ.

Since we used a sparse imaging design with stimuli being presented in-between two consecutive volume acquisitions, gradient artifact removal from the EEG was not necessary (cf. Herrmann and Debener, 2008; Huster et al., 2012). For preprocessing, a finite impulse response (FIR) 100 Hz low-pass filter (389 points, Hamming window) and a 1.7 Hz high-pass filter (4901 points, Hann window, corresponding to a cut-off period of 1/1.7 Hz = 588 ms) was applied to the raw data. Note that filter settings were chosen such that smearing of gradient artifacts into time windows of interest were prohibited. Subsequently, filtered EEG data were down-sampled to 500 Hz and subjected to an independent components analysis (ICA) for artifact correction, using the routines provided by EEGLab (Delorme and Makeig, 2004) and *fieldtrip* (Oostenveld et al., 2011) within MATLAB 7.9 (MathWorks, Natick, MA). Note that the ECG channel was removed prior to ICA analysis. ICAs were calculated on 3-s epochs, with 1 s before and 2 s after stimulus onset. The separation of ICA components (total: 29) representing artifacts from those representing physiological EEG activity was done by visual inspection of the components' time-courses, topographies, and frequency spectra (cf. Debener et al., 2010), using custom-made fieldtrip scripts. Components either showing similar dynamics as the ECG channel or resembling electroocculogram activity as illustrated in Debener et al. (2010) were considered artifacts. Note that it has been observed that ICA-based correction of cardio-ballistic artifacts performs better than standard artifact subtraction methods (Debener et al., 2007; Jann et al., 2009). On average, 7 components were therefore excluded (range: 5–9) by using the ICA-based artifact removal within fieldtrip (Oostenveld et al., 2011).

We furthermore identified bad EEG channels after artifact removal as channels exceeding a threshold of 150 μV in more than 50% of all trials per participant. Bad channels (of which no participant showed more than 1) were interpolated by using signal information from the average of 4–5 neighboring channels (depending on channel location).

In addition to EEG recordings inside the MR-scanner, we tested 18 different participants (9 females, mean age 25, range 20–31 years) outside the scanner. Presenting pre-recorded EPI sounds at times the scanner would have operated simulated the scanner noise. For this control group, the EEG was obtained from 64 Ag-AgCl-electrodes (58 scalp electrodes, 2 mastoids, 2 electrodes for horizontal and 2 for vertical electrooculograms) on a Brain Vision EEG system (amplifier: BrainAmp, cap: BrainCap, Brain Products, Munich, Germany), arranged according to the extended 10/20 system, (Oostenveld and Praamstra, 2001). Otherwise, stimulus presentation, EEG pre-processing and analyses were identical to the procedures described here. However, due to a technical problem with one participant, and more than 30% ICA-artifact components in two further participants, the resulting participant number of the control experiment was 15. This experiment served the purpose of testing the validity of the recordings obtained inside the scanner. Note, however, that overall magnitude differences should not be compared between the experiments inside and outside the scanner, due to different recording equipment.

### Acquisition and pre-processing of fMRI data

Functional MRI data were recorded with a Siemens VERIO 3.0-T MRI scanner equipped with a 12-channel head coil, while participants performed the categorization task in supine position inside the scanner. Acoustic stimuli were transmitted through MR-compatible headphones (mr confon GmbH, Magdeburg, Germany). In-ear hearing protection (Hearsafe Technologies GmbH, Cologne, Germany) reduced scanner noise by approximately 16 dB.

Seventy-five whole-brain EPI volumes (30 axial slices, thickness = 3 mm, gap = 1 mm) in each of the 4 runs were collected every 9 s (TA = 2 s; TE = 30 ms; flip angle = 90°; field of view = 192 × 192 mm; voxel size = 3 × 3 × 4 mm). High-resolution, 3D MP-RAGE T1-weighted scans were used for localization and co-registration (acquired on a 3T Siemens TIM Trio scanner with a 12-channel head coil 29 months prior to the experiment, with the parameters: sagittal slices = 176, repetition time = 1300 ms, TE = 3.46 ms, flip angle = 10°, acquisition matrix = 256 × 240, voxel size = 1 × 1 × 1 mm). Voxel-displacement-maps for distortion correction (Jezzard and Balaban, 1995; Hutton et al., 2002) were calculated on the basis of field maps (30 axial slices, thickness = 3 mm, gap = 1 mm, repetition time = 488 ms, TE1 = 4.92 ms, TE2 = 7.38 ms, flip angle = 60°, field of view = 192 × 192 mm, voxel size = 3 × 3 × 3 mm).

Functional (T2^*^-weighted) and structural (T1-weighted) images were processed using Statistical Parametric Mapping (SPM8; Wellcome Department of Imaging Neuroscience, Institute of Neurology, University College of London). Functional images were first realigned using the 6-parameter affine transformation in translational (x, y, and z) and rotational (pitch, roll, and yaw) directions to reduce individual movement artifacts (Ashburner and Good, 2003). Subsequently, a mean image of each run-based image series was used to estimate unwarping parameters, and voxel-displacement-maps were used for correcting magnetic field deformations (Jezzard and Balaban, 1995; Hutton et al., 2002). Participants' structural images were manually pre-aligned to a standardized EPI template (Ashburner and Friston, 2004) in MNI space, improving co-registration and normalization accuracy. Next, functional images were co-registered to the corresponding participants' structural images and normalized to MNI space. Functional images were then smoothed using an 8-mm full-width half-maximum Gaussian kernel and subsequently used for first-level general linear model (GLM) analyses.

### Analysis of behavioral data

Our behavioral dependent measures were *overall performance* and *cue utilization*. Overall performance was estimated by *d′*, a measure of perceptual sensitivity that is independent of response bias. Perceptual sensitivity, *d′*, was calculated from proportions of hits and false alarms according to a one-interval design (Macmillan and Creelman, 2005), where hits were defined as “category-A” responses to category-A stimuli, and false alarms were defined as “category-A” responses to category-B stimuli. Perceptual sensitivity was calculated separately for each experimental run (2 nondegraded, 2 degraded runs). In order to visualize performance over time, we additionally calculated *d′* values in sliding windows (size: 20 trials, step size: 1 trials), separately for the nondegraded and the degraded condition, and with the exclusion of null trials.

The measure of *cue index* quantified individual participants' cue utilization (spectral peak vs. physical duration) in the following way: First, for each condition, the likelihood of a category-A response was predicted from the stimulus' physical properties, spectral peak and duration, by means of logistic regressions. The slope of the regressions function, expressed by absolute β, indicated the degree to which the corresponding physical stimulus property influenced the categorical response (β_spectral peak_; β_duration_; Goudbeek et al., 2009; Scharinger et al., 2013). Note that β_spectral peak_ and β_duration_ were estimated simultaneously. Second, the normalized difference between these β values (*cue index*) indicated participants' preference to rely on spectral peak (negative values according) or on duration (positive values).

$$\text{Cue}\;\text{index}=\frac{\beta_{\text{duration}-}\;\beta_{\text{spectral}\;\text{peak}}}{\beta_{\text{duration}\text{+}}\beta_{\text{spectral}\;\text{peak}}}$$

### Analysis of EEG data

For the analysis of the event-related potentials (ERPs), single-trial EEG epochs were first re-referenced to linked mastoids (approximated by channels Tp9 and Tp10). Subsequently, epochs were filtered with a 20-Hz Butterworth low-pass filter and re-defined to include a pre-stimulus interval of 500 ms and a post-onset interval of 1500 ms. Baseline correction was applied by subtracting the mean amplitude of the −500 to 0 ms baseline interval from the epoch. Single-trials were averaged separately for the nondegraded and the degraded condition. Auditory N1 components (Näätänen and Picton, 1987) were identified by visual inspection in a time window between 100 and 150 ms post onset. Averaged amplitudes for Cz within the N1 time-window were compared between conditions (nondegraded, degraded) by means of dependent-samples *t*-tests.

For time-frequency analyses, re-referenced EEG-data were down-sampled to 125 Hz and then decomposed with a Morlet wavelets analysis (Bertrand and Pantev, 1994), centered on windows that slid in steps of 10 ms along the temporal dimension (−1 to 2 s). In the spectral dimension, we used 1-Hz bins from 1 to 30 Hz. Wavelet widths ranged from 1 to 8 cycles, equally spaced over the 30 frequency bins. Time-frequency analyses were done separately for nondegraded and degraded trials. Mean power values of a pre-stimulus baseline interval (−500 to −50 ms) were subtracted from the epoch. A time-frequency region of interest (ROI) was chosen according to the typical alpha-band interval (7–11 Hz) and according to epochs that previously showed the suppression effect in speech (400–700 ms post onset, e.g., Obleser and Weisz, 2012; Becker et al., 2013). A consistent and symmetric posterior electrode selection for subsequent EEG/fMRI correlations was based on electrodes where alpha power was strongest in above-mentioned ROI (within the nondegraded condition). These electrodes were: CP1, CP2, P7, P3, Pz, P4, P8, POz, O1, Oz, and O2. Averaged power values in the alpha ROI was compared between conditions by means of dependent-samples *t*-tests.

### Analysis of fMRI data

Activated voxels were identified using the GLM approach (Friston, 2004). At the first level, a GLM was estimated for each participant with a first-order finite impulse response (FIR; window = 2 s) and a high-pass filter with a cut-off of 128 s, representing standard settings for sparse imaging designs (cf. Peelle et al., 2010). The design matrix included regressors for *sound trials* (corresponding to volumes following sound representations), the mean-centered single-trial parametric modulator *alpha power* (obtained from the ROI defined above), and *silent trials* (corresponding to volumes following null trials). Experimental runs were included as regressors of no interest (one for each run). Six additional regressors of no-interest accounted for the realignment-induced spatial deformations of the EPI volumes.

Resulting beta-maps were restricted to gray- and white matter. This information was obtained from group-averages based on individual T1-weighted scans. On the first level, the following contrasts were calculated (separately for nondegraded and degraded conditions): *sound trials* against implicit baseline and parametric modulator *alpha power* against implicit baseline. Furthermore, we calculated the contrasts nondegraded > degraded and degraded > nondegraded.

On the second level (group level), all contrasts were compared against zero using one-sample *t*-tests. Additionally, for each condition (nondegraded, degraded), sound-trial contrasts (against implicit baseline) from the first level were correlated with cue index using linear regression. Differences between nondegraded and degraded conditions in Cue index/BOLD correlation were assessed by testing the slopes of the linear regressions against each other using a dependent samples *t*-test.

For statistical thresholding of second-level activations, we used a threshold of *p* < 0.005 combined with a cluster extent of 15 voxels that corresponds to a whole-brain significance level of *p* < 0.05, as determined from a MATLAB-implemented Monte Carlo simulation (Slotnick et al., 2003; Erb et al., 2013).

In order to visualize BOLD modulation differences across conditions, ROIs of 10 mm radii were defined using the SPM toolbox MarsBaR (Brett et al., 2002). They were centered on the peak coordinates of significant clusters identified in the whole-brain analyses. For these regions, mean regression beta values were estimated for each participant. Note that no additional tests were conducted for these regions to avoid statistical circularity. Determination of anatomical locations was based on the Automated Anatomical Labeling Atlas (AAL; Tzourio-Mazoyer et al., 2002), and PT localization followed Westbury et al. (1999).

## Results

### Behavioral data

Participants performed above chance as indicated by *d′* values significantly greater than zero [mean *d*' = 1.51, *SD* = 0.43; *t*_(14)_ = 19.19, *p* < 0.01]. Participants' performance was characterized by a considerable improvement over the first twenty trials, as estimated from sliding-window averages of *d′*-values (window size: 20 trials, step size: 1 trial, Figure 1B top). After degradation was introduced, performance dropped to the initial level, but quickly regained a stable plateau and did not differ overall from the nondegraded condition [nondegraded vs. degraded *t*_(14)_ = 1.00, *p* = 0.32].

Cue indices marginally differed between conditions [*t*_(14)_ = 1.94, *p* = 0.07], with more negative values for the nondegraded than the degraded condition. This means that the tendency of utilizing spectral cues (i.e., a negative cue index) in the nondegraded condition decreased in the degraded condition (i.e., a positive-going cue index). However, a spectral strategy was never entirely given up, as judged from overall still negative cue indices in the degraded condition (Figure 1B, bottom).

### EEG data

**Figure 2:**
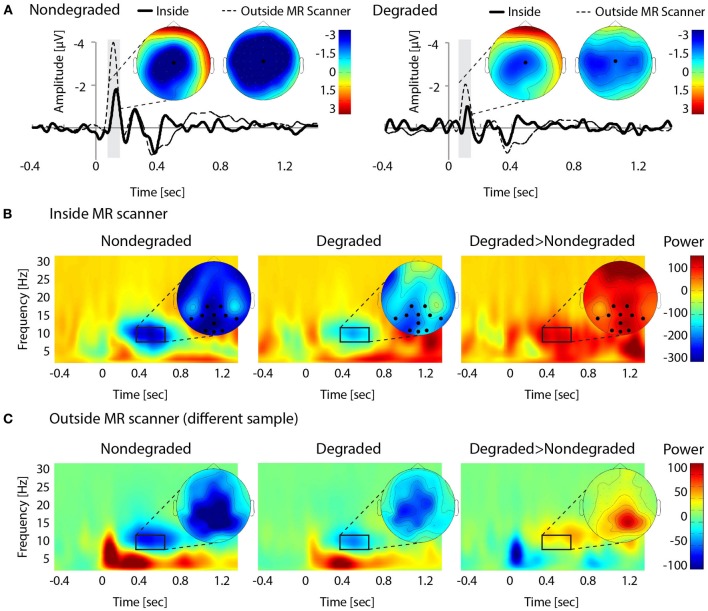
**EEG results. (A)** Grand-average of evoked responses in the nondegraded (left) and degraded (right) condition. ERP-differences between conditions were seen for the N1, with a central/midline distribution (100–150 ms, indicated by gray bars). **(B)** Averaged time-frequency representations for the nondegraded (left) and degraded (middle) condition, and difference between averages (degraded > nondegraded; right). The strongest effect of alpha suppression (compared to baseline) occurred at central-posterior electrodes (selection marked with black dots; 400–700 ms, 7–11 Hz), where it also significantly differed between conditions. **(C)** Averaged time-frequency representations from the control experiment outside the MR scanner (nondegraded: left, degraded: middle, difference: right). Differences and topographies are comparable to within-scanner recordings. Note that overall magnitude differences should not be compared between the experiments inside and outside the MR scanner, due to different recording equipment.

The N1 (100–150 ms) of the ERP showed a typical central/midline topography (inside and outside the scanner). N1 mean amplitude marginally differed between the nondegraded and the degraded condition [*t*_(14)_ = 1.9, *p* = 0.08], with more negative values in the nondegraded than in the degraded condition. This effect reached significance outside the scanner [*t*_(14)_ = 7.89, *p* < 0.01; Figure 2A].

Alpha power (7–11 Hz) around 400–700 ms showed a central-posterior distribution and also differed significantly between conditions, with relatively higher alpha power for the degraded than for the nondegraded condition [*t*_(14)_ = 2.06, *p* = 0.04 Figure 2B]. Again, this effect also held for the control experiment outside the scanner [*t*_(14)_ = 2.56, *p* = 0.03; Figure 2C].

In order to assess the covariation of alpha power and cue index, we calculated correlations between mean alpha power and mean cue index per participant, and in addition, separately for the nondegraded and degraded condition. Overall, mean alpha power and mean cue index did not correlate significantly [*r* = 0.28, *t*_(14)_ = 1.07, *p* = 0.30]. This held both within the nondegraded [*r* = 0.23, *t*_(14)_ = 0.85, *p* = 0.41] and the degraded condition [*r* = 0.16, *t*_(14)_ = 0.60, *p* = 0.56].

### fMRI data

#### Overall auditory categorization network in parietal and temporal areas

**Figure 3:**
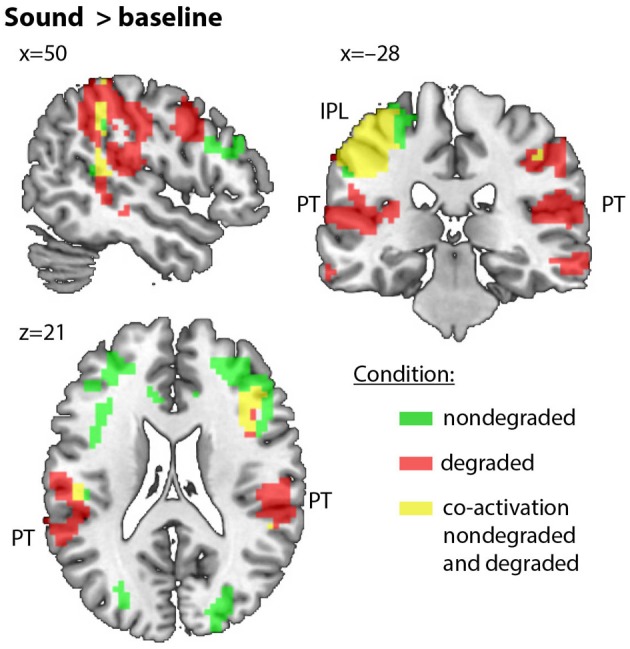
**Regions of the *sounds>baseline* contrast in nondegraded (green) and degraded (red) condition (co-activation in nondegraded and degraded condition: yellow)**. Slices focus on the temporo-parietal network. Note that overlap/co-activation is shown as illustrative means and is not based on statistical measures.

Results from group-level whole-brain analyses showed that the categorization of nondegraded and degraded sounds (compared to baseline) lead to activations in extensive bilateral temporo-parietal clusters, with peaks in inferior parietal lobule and postcentral gyrus (see Figure 3). Furthermore, peaks in precentral and cingulate cortex were predominantly seen for nondegraded sounds, while degraded sounds showed activations in pSTG, PT, and Heschl's gyrus. Both conditions also revealed substantial activations in middle frontal gyrus (MFG), inferior frontal gyrus (IFG), and in the dorsal medial nucleus of left Thalamus.

More activation for degraded than for nondegraded sounds was found in right IFG (extending into the insula), left and right pSTG (including parts of PT, i.e., gray matter with a likelihood of 25–45% being in PT according to Westbury et al., 1999), as well as right STG (extending into the insula). A detailed overview of the clusters is provided in Table 2.

**Table 2:** **Significant clusters obtained from whole-brain analyses (*p* < 0.005, extent threshold = 15) for the contrasts sounds > baseline in each condition, and the contrast degraded sounds > nondegraded sounds**.

**Contrast**	**Area**	**Coordinates**	***Z***	**Extent (voxels)**
Nondegraded sounds > baseline	l. IPL/BA40	−39, −13, 61	4.95	2659
	r. IFG/BA46	45, 38, 31	4.4	539
	r. IPL/SMG	42, −34, 46	4.28	470
	r. Cereb/Culmen	21, −55, −26	4.2	194
	r. Cereb/Culmen	3, −61, −32	4.18	170
	l. Thalamus	−6, −19, 7	3.87	113
	r. Cuneus	18, −91, 1	3.75	103
	l. Insula/BA13	−30, 14, 1	3.66	72
	r. Insula/BA13	30, 20, −2	3.65	61
	r. ITG/BA20	57, −46, −17	3.6	37
	l. Insula/BA13	−27, 26, −5	3.55	21
	l. Occ./BA17	−15, −91, 1	3.49	27
	l. MFG/BA10	−24, 59, −8	3.46	80
	l. pSTG/PT	−48, −46, 7	3.42	30
	r. pSTG/PT	51, −40, 13	3.17	35
Degraded sounds > baseline	l. Postcentral/IPL	−51, −22, 46	5.61	2074
	r. IPL/BA40	39, −43, 58	4.82	1563
	r. Cingulate/BA32	3, 11, 55	4.77	631
	r. Precentral/BA6	48, 5, 40	4.29	354
	l. Cuneus/BA18	−18, −100, 1	4.24	512
	r. MFG/BA11	21, 47, −11	4.24	15
	r. MFG/BA10	36, 50, 10	4	84
	r. IFG/BA47	30, 29, −2	3.7	70
	l. MFG/BA10	−33, 41, 4	3.7	79
	l. MTG/BA21	−63, −31, −14	3.64	41
	l. Thalamus	−12, −19, 10	3.47	63
	l. Insula/BA13	−30, 32, 7	3.32	75
	l. MFG/BA10	−27, 32, 25	3.2	19
	r. Cereb./Culmen	15, −52, −23	3.17	21
Degraded > Nondegraded	r. IFG/Insula	33, 14, −17	3.9	43
	l. pSTG/PT	−51, −37, 10	3.41	16
	r. STG	48, −4, −8	3.3	31
	r. pSTG/PT	54, −25, 19	3.2	30

Abbreviations are explained in the text. Coordinates are given in Montreal Neurological Institute (MNI) space.

#### Alpha power covaries with bold activity in pSTG, PT, and IFG

Group-level whole-brain analyses showed that single-trial alpha power correlated positively with BOLD only in the degraded condition. Here, alpha power/BOLD correlations occurred in two clusters in IFG (comprising pars triangularis and ventral orbitofrontal cortex), in one cluster located in right pSTG (with 25–45% probability of being in PT), and in one cluster in right angular gyrus. In the nondegraded condition, alpha power/BOLD correlations did not survive the statistical threshold.

**Figure 4:**
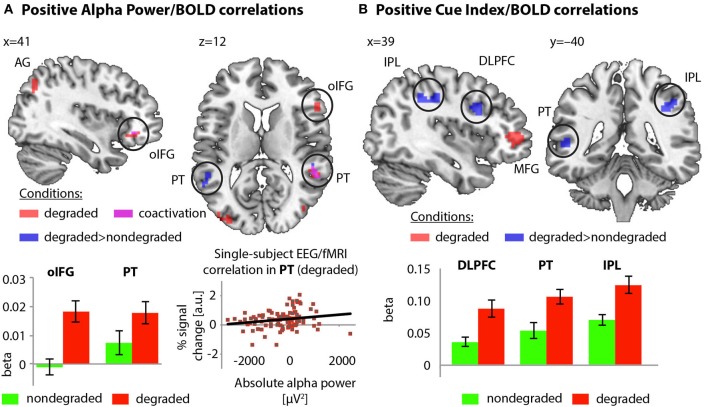
**(A)** Positive correlation of alpha power with BOLD activity in the degraded condition (red), and correlation differences between conditions (blue; co-activation: magenta). Betas extracted from orbital inferior frontal gyrus and PT ROI visualize correlation differences between conditions. Data taken from a representative participant illustrate the positive single-subject alpha power/BOLD correlation in the degraded condition. **(B)** Positive correlations of cue index and BOLD in the degraded condition (red) and correlation differences between conditions (blue). Betas extracted from IFG, dorso-lateral prefrontal cortex and IPL visualize the correlation differences between conditions.

Stronger modulations of BOLD by alpha power could be observed in the orbital part of right IFG, as well as in bilateral pSTG, again comprising parts of the PT (with 25–45% probability according to Westbury et al., 1999; cf. Table 3 and Figure 4A).

**Table 3:** **Significant clusters obtained from whole-brain analyses (*p* < 0.005, extent threshold = 15) for the parametric modulators alpha and cue index, together with modulation differences between conditions**.

**Contrast**	**Area**	**Coordinates**	***Z***	**Extent (voxels)**
Alpha power by BOLD (degraded)	r. oIFG/BA47	45, 29, −8	3.37	49
	r. IFG/BA45	54, 26, 10	3.25	16
	r. pSTG/PT	51, −43, 10	3.14	31
	r. AG/BA39	36, −67, 43	3.04	16
Alpha power by BOLD (nondegraded)	–	–	*n.s.*	
Alpha power degraded > nondegraded	r. oIFG/BA47	45, 29, −11	3.32	18
	r. pSTG/PT	54, −43, 13	3	15
	l. pSTG/PT	−54, −49, 13	2.94	22
Cue index by BOLD (degraded)	r. MFG	39, 47, 4	4.46	58
Cue index by BOLD (nondegraded)	–	–	*n.s.*	
Cue index degraded > nondegraded	r. DLPFC	42, 11, 28	3.74	49
	l. pSTG/PT	−54, −40, 7	3.7	21
	r. IPL	42, −40, 40	3.53	93
	l. MTG	−45, −55, 4	3.28	22

Abbreviations are explained in the text. Coordinates are given in MNI-space.

#### Cue index modulates bold activity in parietal attention and temporal auditory network

Group-level whole-brain regression analyses using the cue index showed positive correlations with BOLD in right MFG (anterior prefrontal cortex) only in the degraded condition. Here, a reduction of using spectral cues corresponded to an increased BOLD signal in anterior prefrontal cortex. By contrast, cue index/BOLD correlations in the nondegraded condition did not survive the statistical threshold.

Furthermore, positive cue index/BOLD correlations were stronger in the degraded than in the nondegraded condition in right dorso-lateral prefrontal cortex (covering parts of pars triangularis and pars opercularis), left pSTG/pSTS (extending into PT), left posterior MTG (involving parts in occipito-temporal cortex), right (ventral) IPL (involving parts of supramarginal gyrus and extending rostrally into postcentral gyrus; cf. Table 3 and Figure 4B).

## Discussion

The two most important findings of this multimodal brain imaging study on auditory categorization are the following: First, auditory categorization of degraded stimuli yielded decreases in alpha power suppression (i.e., relative alpha power increases), which correlated with increased activation in right PT and IFG. Second, even though the behavioral measure of cue utilization only marginally differed between conditions, less reliance on spectral cues under sound degradation corresponded to increased activation in left PT and right IPL. In the subsequent sections, these findings will be discussed in more detail.

### Enhanced alpha power during degraded speech processing

In the current study, categorizing spectrally degraded sounds was accompanied by an attenuation of alpha power suppression. That is, relatively stronger alpha power was observed for the categorization of degraded as compared to nondegraded sounds. This reduction in alpha power suppression (relative to a pre-stimulus baseline) has previously been observed in comparing spectrally degraded speech stimuli to their nondegraded (intelligible) counter-parts (Obleser and Weisz, 2012; Becker et al., 2013). The current data thus extend previous findings by showing that increased alpha power under degradation is not restricted to speech material, but may reflect a more general process that has been interpreted before as enhanced “functional inhibition” (Jensen and Mazaheri, 2010), increased “idling” (Adrian and Matthews, 1934), or a more “active processing state” (Palva and Palva, 2011).

A parsimonious interpretation of this effect relates to the functional inhibition hypothesis of increased alpha power (e.g., Jensen and Mazaheri, 2010). According to this approach, alpha power shows a relative decrease in areas subserving the processing of to-be-attended information (Thut et al., 2006), while it increases in areas subserving the processing of to-be-ignored information (Rihs et al., 2007). Thereby, alpha power dynamics instate a gain mechanism for neural information processing (Jokisch and Jensen, 2007; Kerlin et al., 2010). While the functional role of alpha oscillations in auditory processing and categorization has been examined much less often and only recently (Weisz et al., 2011, 2013; Obleser and Weisz, 2012; Obleser et al., 2012; Becker et al., 2013), the interpretations provided by these previous studies are in line with the functional inhibition hypothesis. For instance, it has been observed that alpha power suppression correlates with the intelligibility of auditory (speech) input (Obleser and Weisz, 2012; Becker et al., 2013). Alpha power suppression was attenuated when auditory stimuli were degraded, that is, when comprehension was more effortful and required higher demands on attention (Obleser et al., 2012), as has been suggested for effortful listening situations before (e.g., Shinn-Cunningham and Best, 2008; Wild et al., 2012).

With respect to our data, we propose that alpha power increases gated the neural processing of acoustic information (duration vs. spectral peak) that differed in task-relevance between conditions: The introduction of spectral degradation in the second half of our experiment changed the relative informativeness or task-relevance of the spectral and duration cues, with spectral peak becoming less informative than stimulus duration. It is thus possible that enhanced alpha under degradation indexed the inhibition of spectral information processing.

Historically, however, enhanced alpha power has first been interpreted as reflecting the degree to which cortical areas are in an “idling” state (Adrian and Matthews, 1934; Niedermeyer and Silva, 2005). Consequently, reduction or suppression of alpha power was taken to index a departure from the idling mode toward a more attentive state. While this interpretation might be applicable for the general suppression of alpha power (vs. baseline) for nondegraded and degraded conditions, it cannot explain the differences in alpha power between conditions. That is, overall performance in our experiment (and thus presumably attentional effort) was comparable between the nondegraded and degraded conditions, while alpha power increased in the latter condition. Thus, this increase in alpha power is unlikely to reflect a more pronounced idling state.

Finally, it has been recently proposed that alpha power enhancement can also be indicative of active processing states (Palva and Palva, 2011). According to the “active processing hypothesis,” enhanced alpha power underlies the coordination of neural processing in task-relevant cortical structures, particularly for higher-order attentional and executive functions. Since the participants in our experiment seemed to be reluctant to refrain from spectral cue utilization under degradation, enhanced alpha power may also relate to “listening” harder for spectral cues, i.e., to an active process of utilizing spectral cues despite their being less informative. Both the “functional inhibition” and “active processing” hypotheses can be applied to the cortical regions in which alpha power positively correlated with BOLD.

### Spectral degradation and the planum temporale

In the degraded condition of our experiment, we observed positive correlations of alpha power with BOLD activations in posterior STG and PT. The posterior STG and the PT have previously been suggested to subserve the processing of spectral information, and in particular, pitch and pitch changes (Zatorre et al., 1994; Zatorre and Belin, 2001; Schönwiesner et al., 2005; Hall and Plack, 2009; Alho et al., 2014). In particular, Hall and Plack (2009) provided evidence that apart from lateral Heschl's gyrus (Schneider et al., 2005; Warren et al., 2005), the (right) PT supports pitch processing to a substantial degree. Importantly, Hall and Plack (2009) used stimuli that bore close resemblance to our degraded sound stimuli such that participants may have perceived and processed pitch differences between our sound categories. Altogether, the involvement of pSTG and PT in our experiment is likely to reflect spectral processing. The positive correlation of alpha power and BOLD activation in this “hub”-like structure for auditory categorization (Griffiths and Warren, 2002) can shed further light onto the relative weighting of spectral vs. duration cues under degradation.

Previous studies using simultaneous EEG-fMRI recordings have observed positive and negative correlations of alpha power with BOLD (Laufs et al., 2003; Gonçalves et al., 2006; de Munck et al., 2007; Goldman et al., 2009; Scheeringa et al., 2009, 2011; Michels et al., 2010; Liu et al., 2012). The interpretation of negative correlations of alpha power with BOLD activations follows the functional inhibition hypothesis (Foxe et al., 1998; Klimesch et al., 2007; Foxe and Snyder, 2011; Weisz et al., 2011, 2013; Klimesch, 2012; Obleser and Weisz, 2012; Obleser et al., 2012). That is, regions where activations increase with decreasing alpha power have been suggested to be relevant for attending to informative stimulus features, while regions where alpha power is positively correlated with BOLD haven been suggested to support the suppression of non-informative (task-irrelevant) stimulus features. Positive correlations of alpha power with BOLD can also be interpreted within the “active processing hypothesis” (Palva and Palva, 2011). This hypothesis relates enhanced alpha power to stronger neural coordination in cortical areas processing task-relevant information, particularly for higher-order attentional and executive functions.

Here, we observed that the posterior STG and the PT showed increased activation for degraded vs. nondegraded stimuli, and that STG and PT activations positively correlated with alpha power. This can either be interpreted with the “functional inhibition hypothesis” or the “active processing hypothesis:”

According to the “functional inhibition hypothesis,” the positive correlation of alpha power with BOLD activation in (right) PT may reflect the relative inhibition of spectral information in this brain area. In detail, introduction of spectral degradation affected the informativeness of spectral peak for categorization, and corresponded to a change in cue utilization. That is, spectral peak became relatively task-irrelevant, and may have been inhibited in pSTG and PT.

According to the “active processing hypothesis,” the positive correlation of alpha power and BOLD activation in pSTG and PT (particularly under degradation) may reflect the enhanced need for neural coordination in order to maintain spectral cue utilization. Overall, cue indices remained negative even after spectral information was degraded, that is, participants still relied on their initial spectral categorization strategy. For maintenance of the spectral strategy, participants might have drawn on (right) posterior STG and PT resources. Thus, the positive correlation of alpha power and BOLD in these cortical regions may index the need to listen “harder” to degraded stimulus cues that once were informative.

Finally, the “active processing hypothesis” seems to receive further support from the positive alpha power/BOLD correlations in frontal (IFG) areas. Note that Palva and Palva (2011) suggest that inhibition at lower sensory levels might be achieved by higher-level frontal functions, such that a positive alpha power/BOLD correlation in IFG may indicate that lesser reliance on spectral than on duration cues under degradation is mediated by activity in frontal regions. This may also relate to the observation that alpha power and behavioral cue utilization indices correlated only at trend-level with each other, suggesting that alpha power changes are more likely reflecting indirect, modulatory signatures of “functional inhibition” (after a stimulus while preparing a response, see also Obleser and Weisz, 2012; Wilsch et al., 2014). These signatures are dissociable from and follow in time early auditory signatures, accounting for the latency of the alpha power effect centered at around 500 ms post stimulus onset.

### A role of the right IPL in auditory attention

The behavioral tendency of disregarding spectral cues in the degraded condition of our experiment was accompanied by increased activation in anterior prefrontal cortex, and, compared to the nondegraded condition, in right IPL. In the degraded condition, right IPL showed a stronger correlation of cue index with BOLD activation than in the nondegraded condition (Figure 4B). As part of the fronto-parietal executive network (Posner and Dehaene, 1994; Corbetta et al., 2000), the IPL has repeatedly been found to subserve selective attention (Shaywitz et al., 2001; Behrmann et al., 2004; Salmi et al., 2009) and attentional control (Hill and Miller, 2010). Its activation was commonly observed in situations that require flexible changes in attention during the processing of informative stimulus features or task-relevant information (Geng and Mangun, 2009; Schultz and Lennert, 2009; Gillebert et al., 2012). In line with studies supporting the IPL's role in selectively attending to the most informative stimulus feature (Jacquemot et al., 2003; Gaab et al., 2006; Husain et al., 2006; Kiefer et al., 2008; Obleser et al., 2012), changes in IPL activation might support the change in cue utilization that was necessary for successful categorization (see Henry et al., 2013 for attention to temporal features). Note however that, behaviorally, participants tried to maintain their initial strategy and overall differed only marginally in cue utilization. Therefore, this interpretation must be considered carefully and substantiated by future research.

## Summary

In this multi-modal imaging study, we have shown that acoustic cue utilization during auditory categorization is flexible, even though listeners seem resilient to abandon initial categorization strategies. Brain areas processing the specific acoustic information—spectral peak vs. duration—supported the change in cue preference together with areas in the fronto-parietal attention network. Our data complement previous speech-related observations of alpha power increases in adverse and effortful listening situations (Obleser and Weisz, 2012; Obleser et al., 2012; Wilsch et al., 2014). We suggest that increased alpha power under degradation mediates the relative weighting of acoustic stimulus features. Both the “functional inhibition” and the “active processing” hypotheses can account for these findings. Importantly, the combination of behavioral, electrophysiological, and hemodynamic measures is an indispensable methodology for further investigations in auditory cognition.

### Conflict of interest statement

The authors declare that the research was conducted in the absence of any commercial or financial relationships that could be construed as a potential conflict of interest.
